# The clinical relevance of oliguria in the critically ill patient: analysis of a large observational database

**DOI:** 10.1186/s13054-020-02858-x

**Published:** 2020-04-23

**Authors:** Jean-Louis Vincent, Andrew Ferguson, Peter Pickkers, Stephan M. Jakob, Ulrich Jaschinski, Ghaleb A. Almekhlafi, Marc Leone, Majid Mokhtari, Luis E. Fontes, Philippe R. Bauer, Yasser Sakr, for the ICON Investigators, Esmael Tomas, Eric Amisi Bibonge, Boubaker Charra, Mamoun Faroudy, Linda Doedens, Zane Farina, David Adler, Cecile Balkema, Adri Kok, Sami Alaya, Hedi Gharsallah, Dritan Muzha, Atanas Temelkov, Georgi Georgiev, Georgi Simeonov, Georgi Tsaryanski, Silvi Georgiev, Ali Seliman, Srdan Vrankovic, Zeljko Vucicevic, Ivan Gornik, Bruno Barsic, Ino Husedzinovic, Pavel Pavlik, Jan Manak, Eva Kieslichova, Radovan Turek, Michal Fischer, Radka Valkova, Lukas Dadak, Pavel Dostal, Jan Malaska, Roman Hajek, Alexandra Židková, Pavel Lavicka, Joel Starkopf, Zurab Kheladze, Mamuka Chkhaidze, Vakhtang Kaloiani, Laszlo Medve, Agnes Sarkany, Ildiko Kremer, Zsuzsa Marjanek, Peter Tamasi, Inga Krupnova, Indulis Vanags, Viesturs Liguts, Vidas Pilvinis, Saulius Vosylius, Gintautas Kekstas, Mindaugas Balciunas, Julia Kolbusz, Andrzej Kübler, Beata Mielczarek, Malgorzata Mikaszewska-Sokolewicz, Katarzyna Kotfis, Barbara Tamowicz, Wiktor Sulkowski, Piotr Smuszkiewicz, Andrzej Pihowicz, Ewa Trejnowska, Natalia Hagau, Daniela Filipescu, Gabriela Droc, Mary Nicoleta Lupu, Alexandru Nica, Radu Stoica, Dana Rodica Tomescu, Dacia Laurentia Constantinescu, Georgica M. Valcoreanu Zbaganu, Adriana Slavcovici, Vladmir Bagin, Dmitry Belsky, Shamil Palyutin, Sergey Shlyapnikov, D. Bikkulova, Alexey Gritsan, Gulyaeva Natalia, Evgeny Makarenko, Vladimir Kokhno, Alla Tolkach, Evgeny Kokarev, Boris Belotserkovskiy, Konstantin Zolotukhin, Vladimir Kulabukhov, Ljiljana Soskic, Ivan Palibrk, Radmilo Jankovic, Bojan Jovanovic, Milena Pandurovic, Vesna Bumbasirevic, Boris Uljarevic, Maja Surbatovic, Nebojsa Ladjevic, Garri Slobodianiuk, Viliam Sobona, Andrea Cikova, Andrea Gebhardtova, Cao Jun, Sun Yunbo, Jun Dong, Sui Feng, Meili Duan, Yuan Xu, Xiaoyan Xue, Tieying Gao, Xue Zhong Xing, Xin Zhao, Chao Hong Li, Gengxihua Gengxihua, Huiqiong Tan, Jingqing Xu, Li Jiang, Qin Tiehe, Qin Bingyu, Qindong Shi, Zheng Lv, Liping Zhang, Liu Jingtao, Zheng Zhen, Zheng Wang, Tie Hua Wang, Liu Yuhong, Qian Zhai, Ying Chen, Chunting Wang, Wei Jiang, Wang Ruilan, Youdai Chen, Huang Xiaobo, Huiqing Ge, Tang Yan, Cui Yuhui, Jiuzhi Zhang, Fu Jian-Hong, Hong Zhu, Feifei Huo, Yushan Wang, Chao Li, Ma Zhuang, Zengxiang Ma, Jian Sun, Liuqingyue Liuqingyue, Mingshi Yang, Jianbiao Meng, Shaolin Ma, Yan Kang, Li Yu, Qianyi Peng, Yu Wei, Wei Zhang, Renhua Sun, Alwin Yeung, Wing Lun Wan, K. Kai Cheuk Sin, Kar Lung Lee, Meri Wijanti, Untung Widodo, Halim Samsirun, Tantani Sugiman, Calcarina Wisudarti, Tinni T. Maskoen, Noritaka Hata, Yoshiro Kobe, Osamu Nishida, Dai Miyazaki, Shin Nunomiya, Shigehiko Uchino, Nobuya Kitamura, Koichi Yamashita, Satoru Hashimoto, Hidetada Fukushima, Nik Azman Nik Adib, Li Ling Tai, Bill Tony, Rodolfo Roman Bigornia, Rodolfo Roman Bigornia, Rodolfo Roman Bigornia, Jose Emmanuel Palo, Somnath Chatterjee, Bee Hong Tan, Andrew Kong, Shirley Goh, Chien-Chang Lee, Chaicharn Pothirat, Bodin Khwannimit, Pongdhep Theerawit, Prapaporn Pornsuriyasak, Annop Piriyapatsom, Ahmed Mukhtar, Ahmed Nabil Hamdy, Hisham Hosny, Ali Ashraf, Majid Mokhtari, Shiva Nowruzinia, Amir Hossein Lotfi, Farid Zand, Reza Nikandish, Omid Moradi Moghaddam, Jonathan Cohen, Oded Sold, Tacla Sfeir, Alaa Yasin Hasan, Dena Abugaber, Habib Ahmad, Tarek Tantawy, Salim Baharoom, Haifa Algethamy, Anas Amr, Ghaleb Almekhlafi, Ramazan Coskun, Murat Sungur, Ahmet Cosar, Bülent Güçyetmez, Oktay Demirkiran, Evren Senturk, Hulya Ulusoy, Hakan Korkut Atalan, Simay Serin, Ismail Kati, Zainab Alnassrawi, Ayesha Almemari, Kalpana Krishnareddy, Sayed Kashef, Asad Alsabbah, Germain Poirier, John C. Marshall, Margaret Herridge, Margaret Herridge, Rosangela Fernandez-Medero, Gerard Fulda, Sharon Banschbach, Juan Quintero, Elizabeth Schroeder, Corinna Sicoutris, Renaud Gueret, Rahul Kashyap, Philippe Bauer, Rahul Nanchal, Richard G. Wunderink, Edgar Jimenez, Andrea Ryan, Denvir Prince, John Edington, Frank Van Haren, Andrew Bersten, David J. Hawkins, Myrene Kilminster, David Sturgess, Marc Ziegenfuss, Stephanie O’ Connor, Jeffrey Lipman, Lewis Campbell, Rick Mcallister, Brigit Roberts, Patricia Williams, Rachael Parke, Patrick Seigne, Ross Freebairn, Daniel Nistor, Chantal Oxley, Paul Young, Ricardo Valentini, Nestor Wainsztein, Pablo Comignani, Maria Casaretto, Giselle Sutton, Paula Villegas, Cayerano Galletti, Jorge Neira, Daniel Rovira, Jorge Hidalgo, Freddy Sandi, Eliana Caser, Marlus M. Thompson, Mariza D’agostino Dias, Luis E. Fontes, Maria C. Lunardi, Nazah C. Youssef, Suzana Lobo, Ricardo Silva, Joao A. Sales, Lina Madeira Campos Melo, Mirella Oliveira, Mariza Fonte, Cintia Grion, Carlos Feijo, Valaria Rezende, Murillo Assuncao, Ana P. Neves, Pablo Gusman, Dyanne Dalcomune, Cassiano Teixeira, Keitiane Kaefer, Israel Maia, Vicente Souza Dantas, Rubens Costa Filho, Fabio Amorim, Maria Assef, Paulo Schiavetto, Joao Houly, Fabiano Bianchi, Fernando Dias, Carla Avila, Leila Rego, Priscylla Castro, Joel Passos, Ciro Mendes, Cintia Grion, Giovana Colozza Mecatti, Marcia Ferrreira, Vivian Irineu, Marcio Guerreiro, Sebastian Ugarte, Vinko Tomicic, Christian Godoy, Wagner Samaniego, Ignacio Escamilla, Ignacio Escamilla, Luis F. Castro Castro, Giovanni Libreros Duque, Diego Diaz-Guio, Frederico Benítez, Arturo Guerra Urrego, Ricardo Buitrago, Guillermo Ortiz, Maria C. Villalba Gaviria, Donat Salas, Jorge Ramirez-Arce, Estuardo Salgado, Diego Morocho, Jose Vergara, Miguel Chung Sang, Carlos Orellana-Jimenez, Lorena Garrido, Oscar Diaz, Dabor Resiere, Carlos Osorio, Alberto De La Vega, Raul Carrillo, Victor Sanchez, Asisclo Villagomez, Ricardo Martinez Zubieta, Maria Sandia, Maycotte Zalatiel, Manuel Poblano, Daniel Rodriguez Gonzalez, Fernando Arrazola, Leal L. Juan Francisco, Silvio A. Ñamendys-Silva, Rosalinda Guerra Moya, Marco Hernandez, Diego M. Rodriguez Cadena, Ines Lopez Islas, Carlos M. Ballesteros Zarzavilla, Alfredo Matos, Indira Oyanguren, Jorge Cerna, Rosario Quispe Sierra, Rocio Jimenez, Luis Castillo, Ramizan Ocal, Atilla Sencan, Silvia M. Mareque Gianoni, Alberto Deicas, Javier Hurtado, Gaston Burghi, Antonio Martinelli, Ingrid Von Der Osten, Christine Du Maine, Mahuya Bhattacharyya, Susruta Bandyopadhyay, Srishar Yanamala, Palepu Gopal, Samir Sahu, Mohamed Ibrahim, Darshana Rathod, Nisha Mukundan, Arun Dewan, Pravin Amin, Srinivas Samavedam, Bhagyesh Shah, Ganavelu Gurupal, Brajendra Lahkar, Amit K. Mandal, Mrinal Sircar, Supradip Ghosh, Veluchamy Balasubramani, Farhad Kapadia, Sonali Vadi, Kesavan Nair, Swagat Tripathy, Sivakumar Nandakumar, Jeetendra Sharma, Arindam Kar, Simant Jha, Kapil Zirpe, Mayur Patel, Ankur Bhavsar, Devi P. Samaddar, Atul Kulkarni, Madiha Hashmi, Wajid Ali, Syed Nadeem, Kanishka Indraratna, Antoni Margarit, Philipp Urbanek, Joachim Schlieber, Johann Reisinger, Johann Auer, Andreas Hartjes, Andreas Lerche, Thomas Janous, Eveline Kink, Walter Krahulec, Karl-Heinz Smolle, Marc Van Der Schueren, Patrick Thibo, Marc Vanhoof, Ibis Ahmet, Philippe Gadisseux, Philippe Dufaye, Olivier Jacobs, Vincent Fraipont, Patrick Biston, Alain Dive, Yves Bouckaert, Eric Gilbert, Benjamin Gressens, Eric Pinck, Vincent Collin, Jean-Louis Vincent, Jan J. De Waele, Rocio Rimachi, Dan Gusu, Koen De Decker, Kakisidi Mandianga, Luc Heytens, Xavier Wittebole, Herbert Spapen, Olivier Van Collie, Wout Vandenheede, Peter Rogiers, Piotr Kolodzeike, Mary Kruse, Torben Andersen, Veli-Pekka Harjola, Kari Saarinen, Marc Leone, Alain Durocher, Serge Moulront, Alain Lepape, Marie-Reine Losser, Philippe Cabaret, Evangelos Kalaitzis, Elie Zogheib, Philippe Charve, Bruno François, Jean-Yves Lefrant, Bassam Beilouny, Xavier Forceville, Benoit Misset, Frederic Jacobs, Bernard Floccard, Didier Payen, Alain Wynckel, Vincent Castelain, Alexandre Faure, Pierre Lavagne, Thierry Lepoivre, Mouhamed D. Moussa, Antoine Vieillard-Baron, Michel Durand, Marc Gainnier, Carole Ichai, Stefan Arens, Clemens Hoffmann, Magnus Kaffarnik, Claus-Jorg Scharnofske, Ingo Voigt, Claus Peckelsen, Matthias Weber, Jochen Gille, Andreas Lange, Georg Schoser, Armin Sablotzki, Ulrich Jaschinski, Andreas Bluethgen, Frank Vogel, Andreas Tscheu, Thomas Fuchs, Michael Wattenberg, Torsten Helmes, Stefan Scieszka, Matthias Heintz, Samir Sakka, Johannes Kohler, Fritz Fiedler, Matthias Danz, Yasser Sakr, Reimer Riessen, Thomas Kerz, Alexander Kersten, Frank Tacke, Gernot Marx, Thomas Volkert, Axel Schmutz, Axel Nierhaus, Stefan Kluge, Peter Abel, Rolf A. Janosi, Stefan Utzolino, Hendrik Bracht, Susanne Toussaint, Maria Giannakou Peftoulidou, Pavlos Myrianthefs, Apostolos Armaganidis, Christina Routsi, Angela Xini, Eleni Mouloudi, Ioannis Kokoris, George Kyriazopoulos, Sawas Vlachos, Athena Lavrentieva, Panagio Partala, George Nakos, Alma Moller, Sigurjon Ö. Stefansson, Joan Barry, Ruth A. O’Leary, Catherine Motherway, Mohammad Faheem, Eimhin Dunne, Maria Donnelly, Torsten Konrad, Eleonora Bonora, Carola Achilli, Sandra Rossi, Giacomo Castiglione, Adriano Peris, Daniela Albanese, Nino Stocchetti, Giuseppe Citerio, Lorella Mozzoni, Erminio Sisillo, Pasquale De Negri, Monica Savioli, Pietro Vecchiarelli, Florin Puflea, Vladimir Stankovic, Giulio Minoja, Silvia Montibeller, Plinio Calligaro, Raffaella Sorrentino, Marco Feri, Massimo Zambon, Elena Colombaroli, Antonino Giarratano, Tommaso Pellis, Carlo Capra, Massimo Antonelli, Antonino Gullo, Cosimo Chelazzi, Antonella De Capraris, Nicolo Patroniti, Massimo Girardis, Frederico Franchi, Giorgio Berlot, Michael Buttigieg, Hubert Ponssen, Julia Ten Cate, Laura Bormans, Satria Husada, Marc Buise, Ben Van Der Hoven, Auke Reidinga, Michael Kuiper, Peter Pickkers, Georg Kluge, Sylvia Den Boer, Jozef Kesecioglu, Henk Van Leeuwen, Hans Flaatten, Skule Mo, Vitor Branco, Fernando Rua, Estevao Lafuente, Marta Sousa, Nuno Catorze, Maria Barros, Luis Pereira, Ana Vintém De Oliveira, Jose Gomes, Isable Gaspar, Maria F. Pereira, Maria Cymbron, Antonio Dias, Eduardo Almeida, Sofia Beirao, Isabel Serra, Rosa Ribeiro, Pedro Povoa, Filomena Faria, Zelia Costa-E-Silva, Jose J. Nóbrega, Fatima Fernandes, Joao Gabriel, Gorazd Voga, Erik Rupnik, Lucka Kosec, Milena Kerin Povšic, Irena Osojnik, Viktorija Tomic, Andreja Sinkovic, Javier González, Elizabeth Zavala, Jesus Pérez Valenzuela, Luis Marina, Pablo Vidal-Cortés, Pilar Posada, Ignacio Martin-Loeches, Noelia Muñoz Guillén, Mercedes Palomar, Jordi Sole-Violan, Antoni Torres, Miguel A. Gonzalez Gallego, Gerardo Aguilar, Raquel Montoiro Allué, Monica Argüeso, Martin Parejo, Manuel Palomo Navarro, Anton Jose, Nicholas Nin, Francisco Alvarez Lerma, Oscar Martinez, Eva Tenza Lozano, Sara Arenal López, Maria J. Perez Granda, Salvador Moreno, Clara Llubia, Carmen De La Fuente Martos, Paloma Gonzalez-Arenas, Noemi Llamas Fernández, Bernard Gil Rueda, Isabel Estruch Pons, Nieves Cruza, Fernando Maroto, Angel Estella, Ana Ferrer, Lisardo Iglesias Fraile, Brigida Quindos, Amaia Quintano, Maria T. Tebar, Pablo Cardinal, Antonio Reyes, Alejandro Rodríguez, Ana Abella, Santiago García Del Valle, Santiago Yus, Emilio Maseda, Jose A. Berezo, Armando Tejero Pedregosa, Clara Laplaza, Ricard Ferrer, Jesus Rico-Feijoo, Marina Rodríguez, Pablo Monedero, Karin Eriksson, Dan Lind, David Chabanel, Hervé Zender, Kuno Heer, Bernd Frankenberger, Stephan Jakob, Alois Haller, Shiju Matthew, Robert Downes, Casiano Barrera Groba, Andrew Johnston, Roseanne Meacher, Rick Keays, Philip Haji-Michael, Chris Tyler, Andrew Ferguson, Simon Jones, David Tyl, Andrew Ball, John Vogel, Malcolm Booth, Paul Downie, Malcolm Watters, Stephen Brett, Marc Garfield, Lynn Everett, Sarah Heenen, Sandeep Dhir, Zoe Beardow, Marthinus Mostert, Steve Brosnan, Nuno Pinto, Stephen Harris, Andy Summors, Andrew Norton, Alastair Rose, Rebecca Appelboam, Omubo Davies, Emma Vickers, Banwari Agarwal, Tamas Szakmany, Stephen Wimbush, Ingeborg Welters, Rupert Pearse, Robin Hollands, Justin Kirk-Bayley, Nick Fletcher, Barbara Bray, David Brealey, Peter Alexander, Steven Henderson, Chris Hargreaves, Heather Black, Kiran Gowda

**Affiliations:** 1 Department of Intensive Care, Erasme University Hospital, Université Libre de Bruxelles, Route de Lennik, 808, 1070 Brussels, Belgium; 2 Department of Intensive Care Medicine, Belfast City Hospital, Belfast, UK; 3 Department of Intensive Care Medicine, Radboud University Medical Center, Nijmegen, The Netherlands; 4 Department of Intensive Care Medicine, University Hospital Bern, University of Bern, Bern, Switzerland; 5 Klinik für Anästhesiologie und Operative Intensivmedizin, Universitätsklinik Augsburg, Universität Augsburg, Augsburg, Germany; 6 ICS Department, Prince Sultan Military Medical City, Riyadh, Saudi Arabia; 7 Service d’Anesthésie et de Réanimation, APHM, Hôpital Nord, Aix Marseille Université, Marseille, France; 8 Department of Internal Medicine, SBMU, Tehran, Iran; 9 Departamento de Medicina Baseada em Evidências, Medicina Intensiva, Urgência e Emergência - Faculdade de Medicina de Petrópolis, Petrópolis, Brazil; 10 Department of Internal Medicine, Division of Pulmonary and Critical Care Medicine, Mayo Clinic, Rochester, MN USA; 11 Departament of Anaesthesiology and Intensive Care, Uniklinikum Jena, Jena, Germany

**Keywords:** Urine output, Renal replacement therapy, Mortality

## Abstract

**Background:**

Urine output is widely used as one of the criteria for the diagnosis and staging of acute renal failure, but few studies have specifically assessed the role of oliguria as a marker of acute renal failure or outcomes in general intensive care unit (ICU) patients. Using a large multinational database, we therefore evaluated the occurrence of oliguria (defined as a urine output < 0.5 ml/kg/h) in acutely ill patients and its association with the need for renal replacement therapy (RRT) and outcome.

**Methods:**

International observational study. All adult (> 16 years) patients in the ICON audit who had a urine output measurement on the day of admission were included. To investigate the association between oliguria and mortality, we used a multilevel analysis.

**Results:**

Of the 8292 patients included, 2050 (24.7%) were oliguric during the first 24 h of admission. Patients with oliguria on admission who had at least one additional 24-h urine output recorded during their ICU stay (*n* = 1349) were divided into three groups: transient—oliguria resolved within 48 h after the admission day (*n* = 390 [28.9%]), prolonged—oliguria resolved > 48 h after the admission day (*n* = 141 [10.5%]), and permanent—oliguria persisting for the whole ICU stay or again present at the end of the ICU stay (*n* = 818 [60.6%]). ICU and hospital mortality rates were higher in patients with oliguria than in those without, except for patients with transient oliguria who had significantly lower mortality rates than non-oliguric patients. In multilevel analysis, the need for RRT was associated with a significantly higher risk of death (OR = 1.51 [95% CI 1.19–1.91], *p* = 0.001), but the presence of oliguria on admission was not (OR = 1.14 [95% CI 0.97–1.34], *p* = 0.103).

**Conclusions:**

Oliguria is common in ICU patients and may have a relatively benign nature if only transient. The duration of oliguria and need for RRT are associated with worse outcome.

## Introduction

Regardless of the exact criteria used to define it, oliguria is often observed in critically ill patients, and yet there are many questions regarding its clinical relevance and impact on outcomes. Reduced urine output can be a physiological response or a reflection of altered tissue perfusion or renal dysfunction [1–3].

Although urine output is now widely used as one of the criteria for the diagnosis and staging of acute renal failure [4–7], there are relatively few studies that have specifically assessed urine output or oliguria as a marker of acute renal failure or outcomes in general populations of intensive care unit (ICU) patients [2, 8, 9]. The clinical importance of oliguria likely depends on its duration. For example, a 1-h period of oliguria during an emergency admission is less important than if the symptom persists for longer periods, when it is more likely to reflect impaired renal function [10, 11].

To provide some global insight into the impact of oliguria and its persistence on outcomes in general ICU patients, we reviewed the large, international Intensive Care over Nations (ICON) database to evaluate the occurrence of oliguria and its association with the need for renal replacement therapy (RRT) and mortality.

## Patients and methods

This study is a substudy of the ICON audit [12]. All adult (> 16 years) patients admitted to a participating ICU (see the list in the “Acknowledgements”) between May 8 and May 18, 2012, were included in the audit, except those who stayed in the ICU for < 24 h for routine postoperative surveillance. Participation was voluntary, with no financial incentive. Ethics committee approval was obtained by the participating institutions according to local ethical regulations.

Data were collected daily for a maximum of 28 days in the ICU and entered using electronic case report forms via a secured internet-based website. Survival data were collected at the time of ICU and hospital discharge, or at 60 days, whichever occurred first. Detailed instructions and definitions were available through a secured website for all participants before starting data collection and throughout the study period. Any additional queries were answered on a per case basis. Validity checks were made at the time of electronic data entry, including plausibility checks within each variable and between variables. Data were further reviewed by the coordinating center for completeness and plausibility, and any doubts clarified with the participating center. There was no on-site monitoring.

Data collection on admission included demographic data and comorbidities. Clinical and laboratory data for the SAPS II score [13] were reported as the worst values within the first 24 h after admission. A daily evaluation of organ function was performed according to the Sequential Organ Failure Assessment (SOFA) score [5]; organ failure was defined as a SOFA subscore > 2 for the organ in question. ICU interventions, including RRT and mechanical ventilation, were recorded daily.

Clinical and microbiologically proven infections were reported daily as well as antimicrobial therapy. Infection was defined according to the criteria of the International Sepsis Forum [14]. Sepsis was defined as the presence of infection with associated organ failure [15]. Septic shock was defined as sepsis associated with cardiovascular failure requiring vasopressor support (SOFA cardiovascular score ≥ 3). The presence of a decision to withhold/withdraw a life-sustaining measure at any time during the ICU stay was also recorded.

On the case report form, investigators recorded urine output in milliliters as a total for each 24-h period. On the day of admission, urine output data were recorded for the period from the time of admission till the start of the next ICU day. For the purposes of this study, oliguria was defined as a calculated urine output < 0.5 ml/kg/h averaged over a 24-h period [2]. To calculate the urine output for the 24-h admission day period, if full urine output data for the first 24 h of admission were recorded, these data were used for the determination of oliguria “on admission.” If data were provided for only *X* hours on the day of admission (because, for example, the patient was admitted at 10 pm and 24-h urine measurement started at midnight so only 2-h urine output were noted), the estimation of urine output on admission was averaged from the data for the *X* hours and the data from the first full day on the ICU (2nd day 24-h urine output/24 × [24 − *X*]). If no data were recorded for the admission day, we were unable to estimate an admission urine output and the patient was not included. The methods of measuring urine output or of assessing body weight were not recorded. Patients with comorbid chronic renal failure were excluded.

For analysis of evolution during the ICU stay, we included only patients who had a urine output recorded on the day of admission and at least one other 24-h urine output value, and separated them into three groups:

- Transient oliguria—oliguria resolved within 48 h after the 24-h admission day
- Prolonged oliguria—oliguria resolved more than 48 h after the 24-h admission day and not present at the end of the ICU stay
- Permanent oliguria—oliguria present on the day of admission and persisting for the whole ICU stay or again present at the end of the ICU stay

### Statistical analysis

Data are shown as means with standard deviation (SD) or 95% confidence intervals (CI), medians and interquartile ranges (IQR), numbers, and percentages. For the descriptive statistics, only available data were used so missing data were subtracted from the denominator when calculating percentages. Differences between groups in distribution of variables were assessed using the analysis of variance (ANOVA), Kruskal-Wallis test, Student’s *t* test, Mann-Whitney test, chi-square test, or Fisher’s exact test as appropriate.

Individual countries were classified into three income groups according to the 2011 gross national income (GNI) per capita, calculated using the World Bank Atlas method [16]: GNI < $4035 = low and lower middle income, GNI $4036–12,475 = upper middle income, and GNI > $12,476 = high income.

To investigate the association between oliguria on admission and mortality, we used a three-level technique with the structure of an individual patient (level 1) admitted to a specific hospital (level 2) within a particular country (level 3). So patients were nested within hospitals within countries. The model includes hospital and country units as random effects to express the concept that patients from the same country and treated in the same hospital share a common environment. The dependency between patients in a hospital within a country is captured through the use of random intercepts. The explanatory variables considered in the model were:

- Individual-level factors: age, sex, SAPS II score, type of admission, source of admission, highest concentration of creatinine, daily fluid balance, mechanical ventilation or RRT at any time during the ICU stay, fluid balance, presence of recorded end-of-life decision, comorbidities, severity of sepsis during the ICU stay, oliguria on admission
- Hospital-level factors: type of hospital, ICU specialty, total number of ICU patients in the previous year, number of staffed ICU beds
- Country-level factors: GNI

Collinearity between variables was checked by inspection of the correlation between them, by looking at the correlation matrix of the estimated parameters, and by looking at the change in parameter estimates and at their estimated standard errors [17]. Q-Q plots were drawn to check for normality in the residuals. The results of fixed effects (measures of association) are given as odds ratios (OR) with their 95% CI. Random effects (measures of variation) measures included the variance (var) and its standard error (se). The restricted maximum likelihood (REML) procedure, which gives unbiased estimates of the model parameters, was used. The Wald test was used to assess the significance of included covariates. The percentage of cases correctly classified and the area under the receiver operating characteristic curve (AUC) are given to quantify the discriminating power of the model. Missing cases for the included variables were analyzed using the missing-value indicator method.

Data were analyzed using IBM® SPSS® Statistics software, version 26 for Windows and R software, version 3.6.1 (CRAN project). All reported *p* values are two-sided, and a *p* value < 0.05 was considered to indicate statistical significance.

## Results

**Fig. 1:**
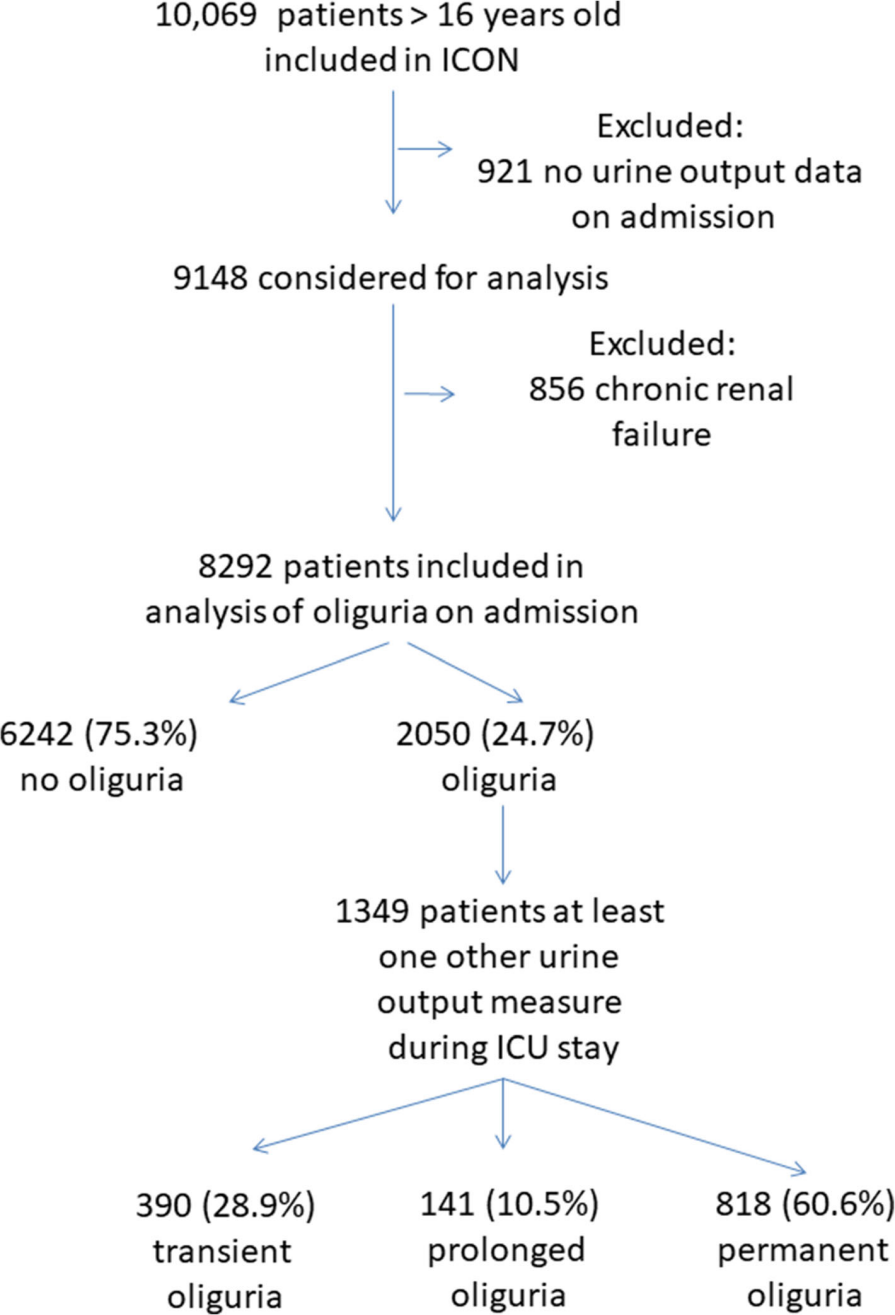
Flow chart of patients included in study

Of the 10,069 patients included in the ICON audit, 9148 had urine output data on the day of admission; 856 had comorbid chronic renal failure and were excluded, leaving 8292 patients for analysis (Fig. 1).

### Patients with oliguria on admission

A total of 2050 (24.7%) patients had oliguria on the 24-h admission period, with a median urine output of 0.3 [IQR 0.1–0.4] ml/kg/h compared to 1.1 [IQR 0.8–1.6] ml/kg/h in patients who were not oliguric on admission (*p* <  0.001) (Table 1). Patients with oliguria on admission were older and more severely ill than those without, and a higher proportion had a medical diagnosis and comorbid heart failure, liver cirrhosis, and human immunodeficiency virus (HIV) infection (Table 1). They were more likely to have sepsis (24.5% vs 16.7%, *p* <  0.001) and to have all forms of organ failure, except hepatic (Table 1). Vasopressor use was higher in patients with oliguria at admission than in those without (36.9% vs 26.8%, *p* <  0.001).

**Table 1:** Characteristics of the study cohort on admission to the ICU stratified according to whether or not oliguria was present

	All patients ***n*** = 8292	Oliguria at admission		***p*** value
		No ***n*** = 6242 (75.3%)	Yes ***n*** = 2050 (24.7)	
Urine output (ml/kg/h) median [IQR]	0.9 [0.5–1.4]	1.1 [0.8–1.6]	0.3 [0.1–0.4]	< 0.001
Age, years, mean ± SD	59.2 ± 18.2	58.1 ± 18.3	62.5 ± 17.4	< 0.001
Male, *n* (%)	4928 (60.0)	3724 (60.2)	1204 (59.4)	0.55
Severity scores, mean ± SD				
SAPS II score	40.8 ± 17.5	37.9 ± 15.1	49.8 ± 20.8	< 0.001
SOFA score at admission	6.2 ± 4.2	5.7 ± 3.8	7.6 ± 5.0	< 0.001
SOFA score at admission (without renal subscore)	5.3 ± 3.7	5.1 ± 3.5	5.8 ± 4.2	< 0.001
Type of admission, *n* (%)				
Surgical (non-trauma)	2939 (37.2)	2374 (39.9)	565 (29.2)	< 0.001
Medical	4335 (54.9)	3063 (51.4)	1272 (65.7)	
Trauma	569 (7.2)	478 (8.0)	91 (4.7)	
Other	48 (.6)	40 (.7)	8 (.4)	
Source of admission, *n* (%)				
Other hospital	825 (9.9)	625 (10.0)	200 (9.8)	< 0.01
ER/ambulance	3151 (38.0)	2336 (37.4)	815 (39.8)	
OR/recovery room	1569 (18.9)	1304 (20.9)	265 (12.9)	
Hospital floor	2117 (25.5)	1529 (24.5)	588 (28.7)	
Other	630 (7.6)	448 (7.2)	182 (8.9)	
Comorbidities, *n* (%)				
COPD	1012 (12.2)	737 (11.8)	275 (13.4)	0.06
Cancer	891 (10.7)	684 (11.0)	207 (10.1)	0.29
Metastatic cancer	288 (3.5)	205 (3.3)	83 (4.0)	0.11
Hematologic cancer	178 (2.1)	126 (2.0)	52 (2.5)	0.16
Insulin	682 (8.2)	496 (7.9)	186 (9.1)	0.12
Heart failure, NYHA III/IV	648 (7.8)	445 (7.1)	203 (9.9)	< 0.001
HIV infection	56 (.7)	33 (.5)	23 (1.1)	< 0.01
Cirrhosis	283 (3.4)	188 (3.0)	95 (4.6)	< 0.001
Immunosuppression	259 (3.1)	184 (2.9)	75 (3.7)	0.11
Steroid therapy	273 (3.3)	208 (3.3)	65 (3.2)	0.78
Chemotherapy	239 (2.9)	181 (2.9)	58 (2.8)	0.94
Organ support, *n* (%)				
Mechanical ventilation	4227 (51.0)	3184 (51.0)	1043 (50.9)	0.92
Renal replacement therapy	321 (3.9)	97 (1.6)	224 (10.9)	< 0.001
Vasopressor use	2429 (29.3)	1672 (26.8)	757 (36.9)	< 0.001
Type of organ failure, *n* (%) (alone or in combination)				
Respiratory	1867 (22.5)	1334 (21.4)	533 (26.0)	< 0.001
Coagulation	530 (6.4)	345 (5.5)	185 (9.0)	< 0.001
Hepatic	885 (10.7)	658 (10.5)	227 (11.1)	0.51
CNS	1972 (23.8)	1435 (23.0)	537 (26.2)	< 0.01
Renal	1341 (16.2)	524 (8.4)	817 (39.9)	< 0.001
Cardiovascular	2296 (27.7)	1580 (25.3)	716 (34.9)	< 0.001
Number of organ failures, *n* (%)				
None	1771 (21.4)	1489 (23.9)	282 (13.8)	< 0.001
1 organ	2344 (28.3)	1805 (28.9)	539 (26.3)	
2 organs	1693 (20.4)	1302 (20.9)	391 (19.1)	
3 organs	1231 (14.8)	883 (14.1)	348 (17.0)	
> 3 organs	1253 (15.1)	763 (12.2)	490 (23.9)	
Sepsis, *n* (%)	1543 (18.6)	1041 (16.7)	502 (24.5)	< 0.001
ICU stay, median [IQR]	3.0 [2.0–6.0]	3.0 [2.0–6.0]	3.0 [1.0–7.0]	< 0.001
In survivors	3.0 [1.0–6.0]	3.0 [1.0–6.0]	3.0 [0.0–7.0]	0.046
In non-survivors	3.0 [0.0–8.0]	5.0 [1.0–10.0]	2.0 [0.0–6.0]	< 0.001
Hospital stay, median [IQR]	10.0 [5.0–20.0]	10.0 [6.0–20.0]	8.0 [2.0–18.0]	< 0.001
In survivors	11.0 [2.0–21.0]	11.0 [2.0–21.0]	11.0 [1.0–22.0]	0.075
In non-survivors	5.0 [0.0–14.0]	7.0 [1.0–15.0]	3.0 [0.0–10.0]	< 0.001
ICU mortality, *n* (%)	1234 (15.2)	700 (11.5)	534 (26.8)	< 0.001
Hospital mortality, *n* (%)	1649 (21.1)	983 (16.7)	666 (34.5)	< 0.001

*SD* standard deviation, *SAPS II* Simplified Acute Physiology Score II, *SOFA* Sequential Organ Failure Assessment, *ER* emergency room, *OR* operating room, *COPD* chronic obstructive pulmonary disease, *NYHA* New York Heart Association, *HIV* human immunodeficiency virus, *CNS* central nervous system, *ICU* intensive care unit, *IQR* interquartile range. Percentages are calculated after excluding missing data

ICU (26.8% vs 11.5%, *p* <  0.001) and hospital (34.5% vs 16.7%, *p* <  0.001) mortality rates were more than twice as high in patients with oliguria on admission than in those without (Table 1). End-of-life decisions were more common in oliguric patients than in the other patients (Table 2).

**Table 2:** Interventions and occurrence of sepsis during the ICU stay

	All patients ***n*** = 8292	Oliguria		***p*** value
		No ***n*** = 6242 (75.3%)	Yes ***n*** = 2050 (24.7%)	
Creatinine, highest concentration (mg/dl), median [IQR]	1.0 [0.8–1.5]	0.9 [0.7–1.3]	1.3 [0.9–2.4]	< 0.001
Daily fluid balance^a^, ml, median [IQR]	81.0 [− 504.1–730.4]	3.3 [− 612–605.7]	356.3 [− 153.2–1116.7]	< 0.001
Mechanical ventilation, *n* (%)	4769 (57.5)	3579 (57.3)	1190 (58.0)	0.59
RRT, *n* (%)	849 (10.2)	407 (6.5)	442 (21.6)	< 0.001
Hemofiltration, *n* (%)	590 (7.1)	268 (4.3)	322 (15.7)	< 0.001
Hemodialysis, *n* (%)	551 (6.6)	266 (4.3)	285 (13.9)	< 0.001
Sepsis severity, *n* (%)				
No sepsis	5718 (69)	4403 (70.5)	1315 (64.1)	< 0.001
Sepsis	1104 (13.3)	858 (13.7)	246 (12.0)	
Shock	1470 (17.7)	981 (15.7)	489 (23.9)	
Decision to withhold/withdraw life-sustaining therapy, *n* (%)	1068 (12.9)	728 (11.7)	340 (16.6)	< 0.001

^a^Total fluid balance divided by the length of ICU stay. *RRT* renal replacement therapy

RRT was needed at some point during the ICU stay in 442 (21.6%) of the patients with and in 407 (6.5%) of the patients without oliguria on admission (*p* <  0.001) (Table 2). Multilevel analysis revealed that the need for RRT was associated with a statistically significant increased risk of death (OR = 1.51 [95% CI 1.19–1.91], *p* = 0.001), but the presence of oliguria on admission was not (OR = 1.14 [95% CI 0.97–1.34], *p* = 0.103) (Table 3). After controlling for patient and hospital factors and GNI, there was significant between-hospital (var = 0.5 [se = 0.09], *p* <  0.001) and between-country (var = 0.23 [se = 0.08], *p* = 0.004) variation in risks of in-hospital death (Table 3), indicating that the occurrence of in-hospital death was influenced by both hospital- and country-related factors.

**Table 3:** Multilevel analysis of factors associated with hospital mortality

Variables	OR (95% CI)	***p*** value
Fixed effects, varying within clusters		
Age	1.00 (1.00–1.01)	0.19
Sex, male	0.99 (0.85–1.14)	0.848
SAPS II	1.05 (1.05–1.06)	< 0.001
Type of admission (%)		
Surgical	Ref	na
Medical	1.53 (1.26–1.86)	< 0.001
Trauma	1.53 (1.14–2.05)	0.004
Other	1.92 (0.71–5.17)	0.196
Source of admission		
OR/recovery	Ref	na
Other hospital	1.17 (0.81–1.68)	0.404
ER/ambulance	1.12 (0.84–1.49)	0.458
Hospital floor	1.65 (1.26–2.16)	< 0.001
Other	1.20 (0.81–1.80)	0.366
Comorbidities		
COPD	0.98 (0.73–1.31)	0.877
Cancer	1.41 (1.15–1.74)	0.001
Metastatic cancer	1.20 (0.86–1.67)	0.281
Hematologic cancer	1.75 (1.25–2.44)	0.001
Insulin	0.84 (0.64–1.09)	0.189
Heart failure, NYHA III/IV	1.54 (1.19–1.99)	0.001
HIV infection	0.73 (0.27–1.94)	0.523
Cirrhosis	2.12 (1.45–3.10)	< 0.001
Immunosuppression	1.19 (0.78–1.79)	0.419
Steroid therapy	1.17 (0.74–1.84)	0.497
Chemotherapy	0.92 (0.55–1.53)	0.748
Creatinine, highest [mg/dl]	1.00 (0.97–1.02)	0.796
Daily fluid balance^a^ [l]	1.37 (1.25–1.50)	< 0.001
Procedures during the ICU stay		
Mechanical ventilation	2.66 (2.12–3.34)	< 0.001
Renal replacement therapy	1.51 (1.19–1.91)	0.001
Severity of sepsis		
No sepsis	Ref	na
Sepsis without shock	0.98 (0.75–1.28)	0.887
Septic shock	1.55 (1.25–1.92)	< 0.001
End-of-life decision	11.82 (6.70–20.84)	< 0.001
Oliguria on admission	1.14 (0.97–1.34)	0.103
Fixed effects, constant within clusters		
Type of hospital		
University/academic	Ref	na
Non-university	1.16 (0.88–1.52)	0.293
Number of patients admitted to the ICU (in 2011)		
750+	Ref	na
500–749	0.95 (0.76–1.19)	0.663
250–499	0.95 (0.68–1.31)	0.746
< 250	1.21 (0.77–1.91)	0.415
ICU specialty		
Surgical	Ref	na
Medical	0.63 (0.38–1.04)	0.072
Mixed	0.73 (0.54–1.00)	0.047
Others	0.94 (0.58–1.52)	0.79
Staffed ICU beds		
15+	Ref	na
< 15	1.12 (0.87–1.44)	0.397
Income		
High	Ref	na
Upper middle	1.81 (1.20–2.73)	0.005
Low and lower middle	2.05 (1.37–3.08)	0.001
Random effects		
Country		
Variance (se)	0.23 (0.08)	
*p* value	0.004	
Hospital within country		
Variance (se)	0.50 (0.09)	
*p* value	< 0.001	

^a^Total fluid balance divided by the length of ICU stay

*OR* odds ratio, *SAPS II* Simplified Acute Physiology Score II, *ER* emergency room, *OR* operating room, *COPD* chronic obstructive pulmonary disease, *NYHA* New York Heart Association, *HIV* human immunodeficiency virus. The percentage of cases correctly classified with this model is 88.6%. The AUC is 91.5% (95% CI 90.7–92.3%)

### Persistence of oliguria during ICU stay

A total of 1349 patients had at least one additional urine output measurement recorded during their ICU stay. Oliguria was transient in 390 (28.9%) of these patients, prolonged in 141 (10.5%), and permanent in 818 (60.6%) (Table 4). ICU mortality was 7.1% when oliguria was transient, significantly lower than in the patients without oliguria (11.5%, *p* = 0.037); rates were 10.9% when oliguria was prolonged and 28.9% when permanent (Table 4).

**Table 4:** Creatinine concentrations, fluid balance, renal replacement therapy, and mortality rates in patients with transient, prolonged, and permanent oliguria

	Non-oliguric on admission ***n*** = 6242	Oliguria during ICU stay, ***n*** = 1349			***p*** value (across groups)
		Transient ***n*** = 390 (28.9%)	Prolonged ***n*** = 141 (10.5%)	Permanent ***n*** = 818 (60.6%)	
SAPS II score, mean ± SD	37.9 ± 15.1	47.1 ± 16.7^£^	54.4 ± 17*^,£^	49.7 ± 18.8^$,£^	< 0.001
Creatinine, highest concentration (mg/dl), median [IQR]	0.9 [0.7–1.3]	1.2 [0.8–2.1]^£^	1.6 [1.1–3.4]*^,£^	1.5 [0.9–2.8]*^,£^	< 0.001
Daily fluid balance^a^, ml, median [IQR]	3.3 [−612–605.7]	49.9 [− 521–650]	105.0 [− 541.3–780.1]	308.9 [− 171.5–1123.7]*^,$,£^	< 0.001
RRT at admission, *n* (%)	97 (1.6)	22 (5.6)^£^	31 (22)*^,£^	121 (14.8)*^,£^	< 0.001
RRT during ICU stay, *n* (%)	407 (6.5)	48 (12.3)^£^	63 (44.7)*^,£^	273 (33.4)*^,$,£^	< 0.001
ICU mortality, *n* (%)	700 (11.5)	27 (7.1)^£^	15 (10.9)	232 (28.9)*^,$,£^	< 0.001
In non RRT patients, *n* (%)	580 (10.2)	22 (6.6)	8 (10.7)	102 (19.1)*^,£^	< 0.001
In RRT patients, *n* (%)	120 (29.9)	5 (10.9)^£^	7 (11.3)^£^	130 (48.3)*^,$,£^	< 0.001
End-of-life decision, *n* (%)	728 (11.7)	47 (12.1)	12 (8.5)	163 (19.9)*^,$,£^	< 0.001
In non RRT patients, *n* (%)	650 (11.1)	43 (12.6)	8 (10.3)	86 (15.8)^£^	0.012
In RRT patients, *n* (%)	78 (19.2)	4 (8.3)	4 (6.3)	77 (28.2)*^,$,£^	< 0.001
Hospital mortality, *n* (%)	983 (16.7)	53 (14.7)	33 (24.8)*	296 (37.7)*^,$,£^	< 0.001

Pairwise *p* values: *vs transient; ^$^vs prolonged; ^£^vs non-oliguric on admission. ^a^Total fluid balance divided by the length of ICU stay. *IQR* interquartile range, *RRT* renal replacement therapy, *ICU* intensive care unit. Percentages are calculated after excluding missing data

RRT was used in the ICU in 48 (12.3%), 63 (44.7%), and 273 (33.4%) patients with transient, prolonged, and permanent oliguria, respectively; 102 (19%) patients with permanent oliguria died in the ICU without receiving RRT. RRT was started within 24 h in 72.4% of patients with oliguria (75.0% vs 73.0% vs 71.8% in patients with transient, prolonged, and permanent oliguria, respectively). Among patients receiving RRT, ICU mortality was higher in non-oliguric patients than in those with transient or prolonged oliguria but lower than in those with permanent oliguria (Table 4). The maximum serum creatinine concentration was higher in patients with prolonged and permanent oliguria than in those with transient oliguria (Table 4). The mean daily fluid balance during the ICU stay was significantly higher in patients with permanent oliguria than in those with transient or prolonged oliguria (Table 4).

## Discussion

The present study in a large cohort of ICU patients with urine outputs measured on admission and during the ICU stay reveals that oliguria is present in about one fourth of critically ill patients on admission to the ICU. The presence of oliguria on admission was not independently associated with an increased risk of death, but the persistence of oliguria during the ICU stay was associated with higher ICU and hospital mortality rates.

There are relatively few published data on the frequency of oliguria in general ICU patients. Oliguria is frequently observed in the perioperative period and may be the consequence of hypovolemia and/or pain, both triggering the sympathetic nervous system, which in turn lead to activation of the renin-angiotensin-aldosterone system with ensuing oliguria. However, oliguria may also represent a warning of deteriorating renal function, especially in critically ill patients. Macedo et al. reported that 47% of their cohort of 317 surgical ICU patients had an episode of oliguria (urine output < 0.5 ml/kg/h for at least 6 consecutive hours) during the ICU stay [1]. In an analysis of data from the FINNAKI study, as many as 92% of patients had an episode of oliguria as defined by a urine output < 0.5 ml/kg/h for a minimum of 0.5 h [3]. From their large database, Kellum et al. reported that 59% of ICU patients with acute kidney injury (AKI) met the KDIGO urine output criteria [8]. In a smaller cohort, Md Ralib et al. reported that 61% of patients with AKI met urine output criteria (< 0.5 ml/kg/h) [11], and in a study of critically ill cancer patients, 56% met urine output criteria for AKI [18].

The mortality rate was higher in patients with oliguria than in those without. In an analysis of 21,207 ICU patients from the large MIMIC-II database, Zhang et al. similarly reported increased mortality in patients with oliguria on day 1 [19]. However, oliguria may be an epiphenomenon of or surrogate for disease severity and after adjusting for multiple factors in the multilevel analysis, oliguria on admission was not independently associated with increased mortality in our patients. Importantly, we also analyzed the persistence of oliguria during the ICU stay and noted that ICU mortality rates in patients with transient oliguria were significantly lower than those in non-oliguric patients. Indeed, ICU and hospital mortality rates were lower in these patients with transient oliguria than in the overall ICON cohort (ICU 16.2%, hospital 22.4%, both *p* <  0.001) [12]. Our results therefore highlight that the duration of oliguria seems to be more important than the presence of oliguria per se. Macedo et al. similarly reported that prolonged duration of oliguria (> 12 h) was associated with increased mortality [1]. Vaara et al. [3] reported that 6–12 h of oliguria (0.3 to < 0.5 ml/kg/h), > 6 h of oliguria (0.1 to < 0.3 ml/kg/h), and severe oliguria (< 0.1 ml/kg/h) lasting > 3 h were independently associated with increased 90-day mortality. In an earlier analysis of the ICON database, patients who remained in stage 3 AKI (defined using the AKIN urine output or creatinine criteria) for a 7-day period had higher mortality rates than those in whom renal function recovered [20]. Prowle et al. [2] noted that although oliguria of longer than 1 h was significantly associated with the subsequent development of AKI diagnosed using creatinine criteria, short periods (1–6 h) of oliguria were not accurate at predicting AKI. In a cohort of patients undergoing major abdominal surgery, the presence of oliguria (urine output < 0.3 ml/kg/h) during surgery was indicative of an elevated probability of later AKI [21]. Similar results were recently published by Myles et al. [22] when a urine output < 0.5 ml/kg/h was used to define oliguria.

Our data also suggest that the increased mortality may be related more to the need for RRT than the oliguria itself, suggesting that other parameters, for example, high serum creatinine concentrations, may be better indicators for RRT than urine output. In an analysis of the MIMIC-II database, Mandelbaum et al. [9] reported that the increase in serum creatinine was a better predictor of the need for RRT than urine output, although urine output was a slightly better predictor of mortality. In their analysis, Kellum et al. reported that RRT use was more likely in patients diagnosed with AKI using urine output and creatinine concentration criteria than in patients diagnosed with AKI using just one of the two criteria [8]. We did not use the AKI criteria, preferring to use the more global term of “acute renal failure” (defined by a renal SOFA score > 2) and need for RRT, because this puts the degree of renal impairment in relation to the dysfunction of the other organs. The SOFA criteria are actually more commonly used than AKI in the critical care literature [23]. Somewhat surprisingly, in the current analysis, RRT was used in only 33% of the patients with permanent oliguria; however, 66% of the patients with permanent oliguria who did not receive RRT were discharged to the hospital floor or another hospital and we have no information about ongoing patient management after ICU discharge. Patients with permanent oliguria were also more likely to have a recorded decision to withhold/withdraw a life-sustaining measure, possibly explaining why RRT was not used in some of these patients.

Recovery of a urine output is not itself a predictive factor, and urine output is not helpful in guiding fluid resuscitation [24]. Patients with permanent oliguria had a more positive fluid balance than those with transient or prolonged oliguria, which may explain in part the higher mortality rates in these patients, although it is not possible to determine whether these observations are epiphenomena or causal effects. Vaara et al. demonstrated an association between cumulative fluid overload (fluid accumulation > 10%) prior to RRT initiation and increased risk for 90-day mortality; the 90-day mortality rate of patients with fluid overload was 59.2% versus 31.4% without (difference of 27.8%, *p* <  0.001) [25]. In an earlier analysis of the ICON database, we reported that fluid balances became negative after the third ICU day in survivors but remained positive in non-survivors and that higher cumulative fluid balance at day 3 after ICU admission was independently associated with an increase in the hazard of death [26]. However, large randomized controlled trials have not shown a significant impact of fluid resuscitation strategy on clinical outcome or need for RRT [27].

Our study has several strengths but also some limitations. Strengths include the large database with patients from around the world, providing external validity, and the collection of data during the ICU stay. Limitations include the complexity of elements associated with oliguria that cannot be separated out, for example, we were unable to assess the need for fluids or diuretics or to assess the impact of different vasopressors. Moreover, criteria for RRT were not pre-defined due to the study design. Thus, RRT may have been used for fluid overload, increased urea and creatinine concentrations, electrolyte abnormalities, severe acidosis, and any combination of these. Another limitation is the lack of pre-admission data regarding the length of oliguria prior to ICU admission or the underlying reason for oliguria as well as the lack of post-discharge data. The methods of monitoring urine output and assessing body weight also likely varied across centers and may have influenced the accuracy of measurements. Finally, we chose a definition of oliguria using a cut-off of urine output of 0.5 ml/kg/h, but this degree of urine output may in fact be adequate for some patients, e.g., the very obese and the very old.

## Conclusion

In conclusion, the present study demonstrates that oliguria is a common occurrence in ICU patients, and suggests that it may have a relatively benign nature if only transient. For prognostic assessment, it is more the duration of oliguria and need for RRT than oliguria per se that are associated with a worse outcome.

## Data Availability

The datasets used and/or analyzed during the current study are available from the corresponding author on reasonable request.

## Abbreviations

AKI: Acute kidney injury
GNI: Gross national income
ICU: Intensive care unit
KDIGO: Kidney Disease Improving Global Outcomes
RRT: Renal replacement therapy
SOFA: Sequential Organ Failure Assessment
